# Chemical Profile and Antioxidant Activity of the Kombucha Beverage Derived from White, Green, Black and Red Tea

**DOI:** 10.3390/antiox9050447

**Published:** 2020-05-22

**Authors:** Karolina Jakubczyk, Justyna Kałduńska, Joanna Kochman, Katarzyna Janda

**Affiliations:** Department of Human Nutrition and Metabolomics, Pomeranian Medical University in Szczecin, 24 Broniewskiego Street, 71-460 Szczecin, Poland; jakubczyk.kar@gmail.com (K.J.); justynakaldunska@wp.pl (J.K.); kochmaan@gmail.com (J.K.)

**Keywords:** kombucha, tea, fermentation, antioxidant, flavonoids, polyphenols

## Abstract

Kombucha is a fermented tea beverage prepared as a result of the symbiotic nature of bacterial cultures and yeast, the so-called SCOBY (*Symbiotic Cultures of Bacteria and Yeasts*). Kombucha is characterised by rich chemical content and healthy properties. It includes organic acids, minerals and vitamins originating mainly from tea, amino acids, and biologically active compounds—polyphenols in particular. Kombucha is prepared mainly in the form of black tea, but other tea types are increasingly often used as well, which can significantly impact its content and health benefits. This work shows that the type of tea has a significant influence on the parameters associated with the antioxidant potential, pH, as well as the content of acetic acid, alcohol or sugar. Red tea and green tea on the 1st and 14th day of fermentation are a particularly prominent source of antioxidants, especially polyphenols, including flavonoids. Therefore, the choice of other tea types than the traditionally used black tea and the subjection of these tea types to fermentation seems to be beneficial in terms of the healthy properties of kombucha.

## 1. Introduction

Unhealthy lifestyle, intense physical exercising, stress, and environmental pollution are factors that influence the excessive synthesis of reactive oxygen species. The disturbance in homeostasis caused by free radicals leads to the formation of oxidative stress and damage to the structures of the human organism [1,2,3,4]. Illnesses that can be caused by free radical disorders include atherosclerosis, neurodegenerative diseases, such as Parkinson’s or Alzheimer’s disease, or even obesity. In order to maintain the balance between the production and removal of reactive oxygen species, it is important to search for easily accessible sources of antioxidants [1]. The main and the most widespread antioxidants are vitamins E, A and C, as well as polyphenolic compounds [1,2,3]. Phenolic compounds are an essential part of the human diet and are of considerable interest due to their health-promoting properties, including antioxidant effects. They are capable of capturing peroxide anions, lipid radicals, hydroxyl radicals and reactive oxygen species. Plant-derived polyphenols have a beneficial impact on slowing down the ageing process and reducing the risk of age-related neurodegenerative conditions, such as Alzheimer’s disease, Parkinson’s disease or ischaemic brain injury [5,6]. Antioxidant sources are mainly searched for in natural plant resources. Antioxidants are present in many easily available sources, such as tea, coffee, fruits, vegetables, spices and herbs. They complement everyday diet, contributing to good health.

Kombucha is a fermented tea drink created with the use of symbiotic cultures of bacteria and yeast, the so-called SCOBY (*Symbiotic Cultures of Bacteria and Yeasts*). Kombucha is prepared by combining tea with sugar (10%), sourdough from previous fermentation (10%) and SCOBY. SCOBY, when added to sugared tea, initiates fermentation, which results in the formation of various new bioactive compounds. The fermentation is conducted at room temperature for a period of 7–14 days. Various tea types can be used to produce kombucha, including green tea, as well as fermented, e.g., red, black or yellow tea. However, black tea and white sugar (saccharose) are considered the traditional and best ingredients that condition the proper content of the drink as well as its healthy properties. The taste of the drink is described as sour, slightly fruity and delicately sparkling, but after a few days of storage it becomes similar to the taste of wine vinegar [7].

Studies of kombucha proved its anti-bacterial, antioxidant, anti-diabetic properties, as well as its ability to reduce the concentration of cholesterol, to support the immune system and to stimulate the detoxification of the liver [8,9]. Kombucha drinks also feature minerals originating mainly from tea (potassium, manganese, fluoride ions), vitamins (E, K, B), amino acids (especially theanine, a derivative of glutamine), as well as other compounds that are formed as the result of numerous reactions occurring during the fermentation of the tea. During the oxidation of polyphenolic compounds, catechins, flavonoids and other compounds with health benefits for the organism are formed [8,10,11].

Various parameters influence the properties and content of kombucha, including the type of tea, fermentation time, the content of SCOBY colonies, and temperature. Despite the increase in the popularity of this drink’s consumption, information regarding the influence of the many parameters or tea types on the properties and content is still not fully available. Hence, the aim of this research was to analyse the antioxidant properties and the content of the drink prepared from black, green, white and red teas at different time points of fermentation [10].

## 2. Material and Methods

### 2.1. Plant Material

The material consisted of four types of leaf tea (*Camellia sinensis*): black Ceylon originating in India, green Gunpowder, white tea and red tea (Pu-ERH) originating in China or India.

### 2.2. Preparation of Kombucha

The kombucha starter cultures, also known as SCOBY (which generally consists of *Acetobacter xylinum*, *Gluconobacter*, *S. cerevisiae*), were obtained from a commercial source from Poland. The starter culture used in the present article was stored in a refrigerator (4 °C) and consisted of sour broth and cellulosic layer (SCOBY floating on the liquid surface). One hundred grams of sugar (100.0 g/L, 10.0%), eight grams of tea (8.0 g/L, 0.8%) and 1 litre of hot, distilled water (90 °C) were mixed. The solution was infused for 10 min in a sterile conical flask. After cooling (30 °C), the tea decoction was filtered through nylon filters (0.45 μm, diam. 25 mm, Sigma-Aldrich, Poznań, Poland) into clean glass bottles. 

### 2.3. Fermentation of Kombucha

Kombucha cultures were kept under aseptic conditions. Fermentation was carried out by incubating the kombucha culture at 28 ± 1 °C for 1, 7 and 14 days. Replicates were prepared so that each replicate was completely collected after its stipulated period of fermentation. The kombucha obtained was filtered and analysed.

### 2.4. Antioxidant Activity by the DPPH Methods

The antioxidant activity of samples was measured with the spectrophotometric method using synthetic radical DPPH (2.2-Diphenyl-1-Picrylhydrazyl, Sigma, Poznań, Poland) according to Brand-Williams et al. and Pekkarinen et al. [12,13]. The spectral absorbance was immediately measured at 518 nm (Agilent 8453UV). All assays were performed in triplicate. The results are shown in % of DPPH radical inhibition. 

Antioxidant potential (antioxidant activity, inhibition) of tested solutions has been expressed by the percent of DPPH inhibition, using the following formula:

$$\%\;\mathrm{inhibition}=\frac{A0-As}{A0}\times 100$$

where:

A0—absorbance of DPPH solution at 518 nm without tested sample

As—absorbance of DPPH solution at 518 nm with tested sample

### 2.5. The Determination of the Ferric Ion Reducing Antioxidant Power (FRAP) Method

The FRAP method, used to determine the total reduction potential, which also means the antioxidant properties of tested ingredient, is based on the ability of the test sample to reduce Fe^3+^ ions to Fe^2+^ ions. The FRAP unit determines the ability to reduce 1 micromole Fe^3+^ to Fe^2+^ according to Benzie and Strain [14,15]. Absorbance at 593 nm was measured (8453UV, AGILENT TECHNOLOGIES, Santa Clara, USA). All assays were performed in triplicate. The ferric ion reducing antioxidant power was determined from the calibration curve using Fe(II)/L as the reference standard (0–5000 µM Fe(II)/L). 

### 2.6. The Determination of the Total Polyphenols Content (TPC)

Determination of polyphenols was performed according to ISO 14502-1; Singleton and Rossi method using the Folin-Ciocalteu reagent [16]. Absorbance at 765 nm was measured (8453UV, AGILENT TECHNOLOGIES, Santa Clara, USA). All assays were performed in triplicate. The content of polyphenols was determined from the calibration curve using gallic acid as the reference standard (0–200 mg/L of gallic acid). 

### 2.7. The Determination of the Total Flavonoids Content (TFC)

Determination of total flavonoids content was performed according to the Pękal and Pyrzynsk and Hu methods [17,18]. Different concentrations of flavonoids were used in the plotting of the standard calibration curve. The content of flavonoids was determined from the calibration curve using rutin equivalent as the reference standard (0–120 mg/L of rutin equivalent). Absorbance at 510 nm was measured (8453UV, AGILENT TECHNOLOGIES, Santa Clara, CA, USA). All assays were performed in triplicate. 

### 2.8. The Determination of pH

The pH of both the fermented beverage and the unfermented control was determined by a pH meter (SCHOTT Instruments; SI Analytics Mainz, Mainz, Germany). 

### 2.9. The Determination of Acetic Acid 

Samples of tea and kombucha at 1, 7 and 14 days of fermentation were filtered through nylon filters (0.45 μm, diam. 25 mm, Sigma-Aldrich, Poznań, Poland). Acetic acid (AA) was analysed by high performance liquid chromatography (HPLC) using a 1200 series HPLC connected to a 1100 series RI detector (Agilent Technologies, Santa Clara, CA, USA) with a Rezex ROA-Organic Acid H^+^ (8%) column (Phenomenex, Torrance, CA, USA). The column was eluted with a degassed mobile phase containing 5 mM H_2_SO_4_, pH 2.25 at 60 °C with a flow rate of 0.5 mL/min for 30 min per sample [19,20]. The results are shown in mg acetic acid/L.

### 2.10. The Determination of Alcohol

The alcohol content was measured using an alcoholometer (Browin, Łódź, Poland). The alcoholometer was immersed in the liquid and the result was read from the scale.

### 2.11. The Determination of Sugar Content

The total sugar content was measured with a laboratory refractometer RL3 (Polish Optical Works, Warsaw, Poland) from Brix scale.

### 2.12. Statistical Analysis

In all the experiments, three samples were analysed, and all the assays were carried out at least in triplicate. The statistical analysis was performed using Stat Soft Statistica 13.0 and Microsoft Excel 2017 (StatSoft Polska, Poland. The results are expressed as mean values and standard deviation (SD). To assess the differences between the examined parameters, the Tukey post hoc test was used. Differences were considered significant at *p* ≤ 0.05. To control type I errors, the false discovery rate (FDR) approach was used. The calculations were performed using the p. adjust function of the stats package in R (R Foundation for Statistical Computing, Vienna, Austria).

## 3. Results

### 3.1. The Analysis of the Antioxidant Properties of Kombucha

The analysis of the antioxidant potential of the studied samples revealed that the content of antioxidant compounds was in the range between 70.62% and 94.61% DPPH inhibition (Table 1). The time of fermentation and the type of tea had an influence on the anti-radical properties of kombucha. In terms of the type of tea, kombucha prepared from green tea was characterised by the highest antioxidant potential, achieving the highest value on the first day of fermentation. In the case of each of the analysed kombucha drinks, the ability to deactivate free radicals decreased with the increase in the time of fermentation.

The highest content of reductive compounds labelled by the FRAP method was observed in all tea types before the fermentation process (5374.1–4486.7 µM Fe(II)/L). The addition of sourdough caused a rapid decrease in the reductive properties of kombucha (3626.3–2274.0 µM Fe(II)/L), but after 7 days of fermentation, the potential increased (4801.1–2725.9 µM Fe(II)/L), then it became lower on the 14th day of fermentation (3172.9–1573.9 µM Fe(II)/L). When analysing the type of the selected tea, kombucha made from green tea was characterised by the highest reductive potential (Table 1).

The analysis of the total content of polyphenols in kombucha, as well as the tea types used for its preparation, revealed that the content of compounds belonging to this group fluctuated in the range from 183.12 mg/L in black tea before the addition of sourdough and SCOBY to 320.12 mg/L in kombucha prepared from green tea on the 14th day of fermentation. In the case of kombucha from green, red and white teas, the highest polyphenol content was observed on the 14th day of fermentation. In kombucha made from green and white tea, the concentration of polyphenolic compounds increased proportionally with the increase in the duration of fermentation. The content of flavonoids, a compound from the group of polyphenols, was the highest for all tea types before starting the fermentation process (395.93 mg/L in red tea). The addition of sourdough significantly reduced flavonoid content in the analysed samples. The decrease in flavonoid content was progressing, achieving the lowest values on the 7th day of fermentation. During the next labelling (14th day of fermentation), there was another increase in the content of this compound (Table 1). In the case of most of the studied parameters, statistically significant differences were observed between tea types as well as the time of fermentation (Table 1).

Table 2 presents the statistically significant correlations between polyphenol content, flavonoids, antioxidant potential (DPPH, FRAP) and the duration of fermentation. Statistical analysis of the results showed significant correlations between the parameters characterising the kombucha antioxidant potential. It was shown that the correlations between the tested parameters are very different, depending on the type of tea (Table 2).

### 3.2. The Analysis of pH, Content of Acetic Acid, Sugar and Alcohol in Kombucha

During the analysis of pH values, it was observed that the pH of all of the studied samples decreased with the increase in the duration of fermentation and the increase in the content of acetic acid. The rapid decrease in this parameter (1.8 unit in the case of kombucha prepared from black tea, up to 2.97 in the case of white kombucha) was caused by the addition of sourdough and SCOBY culture (1st day of fermentation). Further fermentation did not have a significant influence on the change in pH values. No significant differences were observed in terms of pH between drinks prepared from different tea types (Table 3).

With time, the acetic acid content of the fermentation increased, regardless of the type of tea used to prepare kombucha. On the 14th day of fermentation, acetic acid concentration was the highest for all tested beverages (9071.02–9147.40 mg/L) (Table 3). 

The refractometric analysis of sugar content showed that all of the tea types were characterised by the highest concentration of saccharose before the beginning of the fermentation process. When it comes to kombucha prepared with the use of black or white tea, with progressing fermentation the content of saccharose decreased, achieving the lowest value on the 14th day of fermentation (7.5 and 9.5 °Bx, respectively). However, in the case of kombucha made from red and green tea types, the content of saccharose decreased directly after the addition of sourdough, increasing and approaching initial values at the moment of measurement on the 7th day of fermentation. The continuation of the process caused a slow decrease in the content of saccharose in these samples (Table 3).

The concentration of alcohol increased with time, achieving maximum value on the 7th day of fermentation—from 3.0% to 3.5% depending on the tea type. Subsequently, a decrease in alcohol content was observed in all types of kombucha drink (14th day of fermentation).

In the case of most of the studied parameters, statistically significant differences were observed between the time of fermentation. The smallest statistically significant differences were observed between kombucha drinks prepared from various tea types using the same fermentation time (Table 3). Statistical analysis of the results showed significant correlations between the parameters characterising the basic chemical composition of kombucha (Table 4).

## 4. Discussion

The popularity of fermented drinks is increasing as consumers perceive fermentation as a mild method for the preservation of food and value the products themselves for their health benefits. Kombucha, as a fermented tea drink, is consumed not only in Asia, where it originally comes from, but also increasingly often in Europe. It is mainly formed from black tea, but other forms of kombucha made from different tea variants, such as green, white or red tea, are becoming increasingly available on the market. Despite the fact that kombucha has been researched in detail in terms of its microbiological content and antibacterial properties, there are not enough studies regarding the various tea types and their health benefits. This is why our study includes different, most frequently consumed tea types (black, green, white and red) and this is why we analysed the content, antioxidant potential depending on the time of fermentation and the type of tea selected for the preparation of kombucha.

This study has demonstrated that the health benefits as well as the chemical content depend both on the type of tea as well as fermentation time. Kombucha is characterised by high antioxidant potential. Green tea was characterised by the most significant antioxidant properties, slightly lower potential was observed for red and white tea types, whereas black tea featured the lowest values. The same tendency was observed for kombucha prepared from a given tea type. In the case of DPPH, the fermentation process had an influence on the increase in antioxidant properties in reference to tea, and with subsequent days of fermentation the potential decreased regardless of the tea type. A reverse situation was observed in the case of the reductive potential (FRAP). Fermentation had an influence on the decrease in reductive properties with reference to tea. The highest reductive potential was observed for kombucha on the 7th day. Therefore, a strong positive correlation was observed between the time of fermentation and the reductive potential (FRAP) as well as polyphenol content. On the other hand, a negative correlation was observed between the time and the antioxidant potential (DPPH). The differences in the antioxidant potential measured by FRAP and DPPH methods are due to different mechanisms of both methods. In the latter method, the DPPH radical uses the free electron transfer reaction, and the FRAP method utilizes metal ions for oxidation. Additionally, the DPPH method does not allow for the determination of hydrophilic antioxidant activity. FRAP was primarily used to determine the absolute reduction in body fluid. Recently, it has also been adapted for plant-based antioxidant research. In our study, both methods showed high reproducibility. However, the DPPH method has been shown to be more stable [21].

These results are similar to those achieved by Gaggia et al. in a study where the highest antioxidant potential (DPPH) was observed in relation to green tea, slightly lower for white tea, and the lowest for red tea. However, in this case, the 7th day of fermentation had the most positive influence on this parameter. It should be highlighted that the authors did not study kombucha on day one. In all cases, the fermentation process increase the antioxidant properties of the drink [19]. An increase in the antioxidant potential of kombucha in comparison to tea was also observed in the study by Chakravorty et al. [22]. The DPPH and ABTS (2,2’-Azino-bis-3-ethylbenzthiazoline-6-sulphonic acid) radicals’ scavenging activity increased by 39.7% and 38.36%, respectively, after 21 days [22]. It was also observed that the microbiological content is the most diverse on the 7th day of fermentation. This might indicate that the increase in the diversity of microorganisms plays a significant role in the increase in the antioxidant properties of kombucha tea. Moreover, the change of the domination of yeast to lactic acid bacteria on the 7th day is also responsible for the increased antioxidant activity [22]. 

Tea, which is also the main ingredient of the drink, is rich in catechins-theaflavin and tearubigin. Polyphenols present in tea are responsible for the antioxidant activity of kombucha. A positive correlation was observed between the content of polyphenols and reductive potential. This study confirms the observations carried out by Chakravorty et al., in which an increase in polyphenols was observed during fermentation [22]. During fermentation, there is an increase in polyphenols, including flavonoids, whereas tearubigin is transformed into theaflavin, resulting in the change in kombucha’s colour from dark to light with the progressing time of fermentation [22]. On the basis of our studies, it can be concluded that the general content of polyphenols depended on the type of tea. The highest concentration was observed for green tea, slightly lower for red and white tea types, the lowest for black tea. Fermentation time had an influence on the increase in the content of these compounds. Furthermore, an increase in the content of polyphenols in kombucha in comparison to tea alone has also been observed. For kombucha prepared from green and black tea types, the content of polyphenolic compounds increased with the time of fermentation, achieving the highest concentration on the 14th day. Our studies confirm those of other authors. The highest antioxidant potential was also observed in green tea, but on the 7th day of fermentation (100.33 mg/g). Kombucha prepared from red tea included the smallest amount of polyphenols, but they were stable and their concentration did not change during the fermentation process. This kombucha included a lot of flavonoids [19]. The increase in the content of polyphenolic compounds can be associated with numerous reactions occurring during the fermentation of tea, e.g., the oxidation of polyphenolic compounds by some enzymes leads to the formation of catechins, flavonoids and other compounds with healthy properties, including antioxidant properties, which is the result of a microbial hydrolysis reaction [10]. Moreover, microorganisms such as *Candida tropicalis* are able to degrade various polyphenols [23]. Catechins included in the tea can be broken down through the activity of bacteria and yeast into simpler particles, increasing antioxidant strength [10,24]. In addition, fermentation induces the structural breakdown of plant cell walls, leading to the liberation or synthesis of various antioxidant compounds. These antioxidant compounds can act as free radical terminators, metal chelators, singlet oxygen quenchers or hydrogen donors. The production of protease, α-amylase and some other enzymes might be influenced by fermentation possessing metal ion chelation activity [25].

Our study provides an extensive body of evidence that red tea and kombucha are good sources of polyphenols, among them flavonoids with undisputed antioxidant effects Additionally, they helps seal blood vessels, have anti-inflammatory properties and support immune system function [26]. Flavonoids, present in large quantities in red tea, can significantly contribute to its antioxidant properties. A good source of flavonoids also seems to be green tea and kombucha prepared from this variant. However, fermentation contributes to the degradation of this compound. Its highest concentration in red tea subjected to fermentation was observed on day 1 and day 14: 292.54 and 242.5 mg/L, respectively. The value for tea alone was 395.9 mg/L. In comparison, buckwheat—considered as one of the best sources of flavonoids—contains 62.30 mg/100 g of fresh weight of the resource. The tea drink available on the market included only 1.968 mg/L of the resource. Out of 14 of the studied infusions from various tea types, green tea included the highest content of flavonoids—37.13 mg/L [27].

Lactic fermentation is responsible for the breakdown of glucose, which results from the activity of bacteria of lactic fermentation. Another fermentation type is alcoholic fermentation. Yeast, a constituent of the drink’s microflora, is responsible for the breakdown of glucose into ethyl alcohol with the appearance of carbon dioxide. Yeast consists of *Schizosaccharomyces pombe*, as well as *Candida krusei* and *Issatchenkia orientalis* [8]. In our study, on day 7, the highest concentration of alcohol was achieved with as much as 3.5% for kombucha prepared from white and red tea types, 3.25% for green tea and 3.0% for black tea. On the 14th day the content of alcohol slightly decreased in the case of all of the studied variants to the level of 2–3%. In a study by Gaggia et al., the content of alcohol on day 14 was higher—at the level of 5.83% for white tea, 4.18% for green tea and only 1.14% for black tea, but this depends on the fermentation conditions, such as temperature or microbiological composition [19]. In the next phase, *Acetobacter* bacteria [8] use ethyl alcohol as a substrate to create acetic acid. The dominating bacteria included in kombucha are the bacteria of acetic acid AAB: *Acetobacter xylinoides, Acetobacter aceti, Acetobacter pasteurianus, Bacterium gluconicum and Gluconobacter oxydans*. This is why on the 14th day of the fermentation process, the content of alcohol decreased, and there was an increase in acidity as well as the production of organic acids, including acetic acid. Acetic acid, which is the dominating acid present in a fermented solution, contributes to the decrease in pH from 5 to as low as 3 [10,28]. 

An important parameter that undergoes change during fermentation is pH and acidity, and thus the content of organic acids. The microorganisms present in SCOBY process the substances included in tea and sugar, producing various metabolites. This is why these parameters change with fermentation time. In this study, the pH of teas was from 5.34 to 6.53. In the case of kombucha, there was a significant decrease in this parameter: from 2.31 to 2.53 on the 14th day of fermentation. There was also a small decrease in pH between the 7th and 14th day of fermentation, which indicates that the reactions responsible for the decrease in this parameter were inhibited. Our results are similar to the findings of other authors [29,30,31]. Chakravorty et al. observed that the initial pH before fermentation was about 5.03 and decreased abruptly to 2.28 after 7 days of fermentation [22]. It has to be remembered that consuming drinks with a very low pH may negatively influence the digestive system [32]. This is why the fermentation time of kombucha is important, as well as the amount of the consumed drink.

The organic acids present in kombucha include acetic, glucuronic, gluconic, tartaric, malic, citric, lactic, succinic and malonic acids [8,10]. The biochemical content of the drink may slightly differ due to the change of parameters, such as: the amount of sugar, the type and quantity of tea, temperature, pH and the time of fermentation. In this study, for all kombucha types, there was a significant increase in the content of acids during fermentation. The sudden production of organic acids occurred after the 7th day of fermentation. The content of acetic acid on the 14th day of fermentation was the highest for green tea (9147.40 mg/L) and white tea (9132.20 mg/L), the lowest for red tea (9071.02 mg/L) and black tea (9083.03 mg/L). These results correspond to those present in other studies. The research showed differences in metabolite content between the drinks prepared from black tea, green tea and rooibos on different days of fermentation [19]. The content of acetic acid on the 7th day of fermentation present in a study by Gaggìa et al. was the highest in white tea (9.18 mg/mL) and green tea (7.65 mg/mL), while the lowest in rooibos (4.89 mg/mL) [19]. Shahbazi et al. determined that acetic acid was the main acid present in kombucha, and its content significantly decreased during fermentation [29]. Chen and Liu (2000) observed that the concentration of acetic acid increased to 8000 mg/L at the end of the storage period Jayabalan et al. (2007) studied the changes in organic acids of kombucha tea during fermentation [10,33]. They observed that green tea was characterised by the highest content of acetic acid (9500 mg/L) on the 15th day of fermentation [10]. The concentration of lactic acid significantly increased during fermentation. Its concentration was at the level of 145.71 mg/L on the 16th day of fermentation [29]. Malbaša, Lončar and Djurić (2008) used molasses as a source of sugar for the fermentation of kombucha. The content of lactic acid was from 0.16 to 0.4 g/L [34]. It is also worth highlighting that the pH of the solution and the presence of some organic acids determines the growth of microorganisms, and so also the chemical content of the drink [19]. Low pH and high acidity enable the growth of only those microbes that are able to colonise such a niche, so those that can provide a certain kind of protection against unwanted microorganisms [35].

Sugar content in kombucha also changes in time and depends on fermentation. The initial increase in reducing sugar content can be attributed to the hydrolysis of saccharose into glucose and fructose by yeast. With the progressing fermentation, yeast uses sugar in an oxygen-free way to produce ethanol [10]. In our study, the content of sugar decreased with the time of fermentation. The highest decrease (32%) was observed for black tea vs. kombucha on the 14th day of fermentation. Gaggìa et al. checked the content of glucose, fructose and saccharose in kombucha prepared from black, green and red tea types on the 7th and 14th day of fermentation. The content of complex carbohydrates, i.e., saccharose, decreased during fermentation, while the content of simple carbohydrates–glucose–increased. The concentration of fructose increased during fermentation. The highest content of sugars on the 14th day of fermentation was observed in kombucha prepared from red tea [19]. 

In this study, a strong positive correlation was observed between time, acetic acid and pH, whereas a negative correlation was observed between acetic acid and the content of alcohol and sugar. The observed correlations confirm the changes occurring in kombucha during the process of fermentation. The increase in acidity and pH with the time of fermentation, as well as the decrease in alcohol and sugar content are associated with the production of organic acids and the use of substrates for their production.

Kombucha has many health-promoting properties, including antioxidant ones. Therefore, to support one’s antioxidative response, a regular diet should include kombucha, especially in cases of increased exposure to mental and physical stress. Considering the antioxidant properties of kombucha, the most valuable one is derived from red and green tea. However, longer fermentation leads to a decrease in the pH of the drink, which is why consumption of kombucha should be avoided by people suffering from ulcers or gastrointestinal reflux. Of note, kombucha may contain lead from an inadequate vessel, which may be another health hazard [36,37]. 

## 5. Conclusions

Kombucha, the fermented tea, has strong antioxidant properties associated with high polyphenol content, particularly flavonoids. Therefore, it should be consumed by people particularly exposed to oxidative stress. The antioxidant activity of kombucha is diverse and depends on the type and composition of the tea infusion before fermentation and on the content of SCOBY, which determines the character of the forming metabolites and conditions the type of the forming products of polyphenol compound transformation. A particularly rich source of antioxidants, especially flavonoids, are red and green tea types on the 1st and 14th day of fermentation. Therefore, the selection of tea other than black tea, and the subsequent subjection to fermentation, are beneficial to human health.

## Figures and Tables

**Table 1:** Antioxidant potential: DPPH (2.2-Diphenyl-1-Picrylhydrazyl) free radical method, ferric ion reducing antioxidant power (FRAP)), the total polyphenols content (TPC) and total flavonoids content (TFC) in Kombucha tea.

Type of Beverage	Time Points (Day)	Total Flavonoids Content (TFC) [mg/L]	DPPH [%]	FRAP [µM Fe(II)/L]	Total Polyphenols Content (TPC) [mg/L]
**Green Tea Kombucha—GK**	**tea**	254.1 ± 8.6 *^,2,3,4,5^	80.33 ± 2.00 *^,2,3,4^	5374.1 ± 62.1 *^,1,2,3,5,9,13^	269.0 ± 0.9 *^,3,4,5,9,13^
	**1**	196.2 ± 2.6 *^,1,3,4,10,14^	94.61 ± 1.29 *^,1,3,4,6,10,14^	3626.3 ± 36.8 *^,1,3,4,6,10,14^	277.6 ± 0.4 *^,3,4,6,10,14^
	**7**	146.8 ± 3.4 *^,1,2,4,7,11,15^	91.40 ± 0.57 *^,1,2,4,7,11,15^	4801.1 ± 69.2 *^,1,2,7,11,15^	299.6 ± 3.1 *^,1,2,4,7,11,15^
	**14**	181.3 ± 4.8 *^,1,2,3,8,12,16^	88.23 ± 0.83 *^,1,2,3,8,12,16^	3172.9 ± 379.7 *^,1,2,8,12,16^	320.1 ± 3.5 *^,1,2,3,8,12,16^
**Black Tea Kombucha—BK**	**tea**	231.7 ± 11.0 *^,1,6,7,8,9,13^	70.40 ± 0.78 *^,1,6,8,9,13^	4486.7 ± 65.0 *^,1,6,7,8,9,13^	183.1 ± 2.3 *^,6,7,8^
	**1**	149.1 ± 0.6 *^,2,5,7,8,10,14^	78.62 ± 0.63 *^,2,5,7,8,10,14^	2274.0 ± 36.2 *^,2,5,7,10,14^	201.0 ± 5.7 *^,5^
	**7**	90.5 ± 0.7^*5,6,8,11^	70.63 ± 0.53 *^,6,3,11,15^	2725.9 ± 41.0 *^,5,6,8,7,11,15^	219.5 ± 2.1 *^,5,3,7,15^
	**14**	126.7 ± 5.2 *^,5,6,7^	61.04 ± 1.99 *^,4,5,6,7,12,16^	1573.9 ± 182.1 *^,4,5,7,12,16^	206.0 ± 1.2 *^,5,4,6,12^
**White Tea Kombucha—WK**	**tea**	209.3 ± 3.1 *^,5,10,11,12^	78.55 ± 0.35 *^,10,12,1^	4890.0 ± 8.90 *^,1,5,10,11,12,13^	184.6 ± 2.0 *^,10,11,12^
	**1**	132.6 ± 4.8 *^,2,6,9,11,12,14^	89.01 ± 0.99 *^,9,11,12^	2555.4 ± 26.2 *^,2,6,9,11,12,14^	200.8 ± 7.6 *^,9,10,11^
	**7**	83.8 ± 3.3 *^,7,9,10,12^	79.13 ± 0.93 *^,9,10,12,3,7,15^	3263.8 ± 46.3 *^,3,7,9,10,12,15^	205.6 ± 3.0 *^,3,7,9,10,12,15^
	**14**	111.6 ± 2.2 *^,9,10,11^	70.42 ± 1.38 *^,9,10,11,16^	2290.6 ± 171.0 *^,4,8,9,10,11,16^	228.1 ± 0.5 *^,4,8,9,10,11,16^
**Red Tea Kombucha—RK**	**tea**	395.9 ± 2.0 *^,1,5,9,14,15,16^	78.54 ± 0.06 *^,5,14,16^	5261.9 ± 26.5 *^,1,5,9,14,15,16^	229.5 ± 2.9 *^,15,16,1,5,9^
	**1**	292.5 ± 2.3 *^,2,6,10,13,15,16^	89.56 ± 0.08 *^,13,15,16^	2704.6 ± 7.3 *^,2,6,10,13,15,16^	219.8 ± 22.8 *^,15,16^
	**7**	198.1 ± 2.9 *^,3,7,11,13,14,16^	77.37 ± 0.80 *^,3,7,11,14,16^	4314.3 ± 53.5 *^,3,7,11,13,14,16^	270.5 ± 2.4 *^,3,7,11,13,14^
	**14**	242.5 ± 4.8 *^,4,8,12,13,14,15^	74.78 ± 2.11 *^,12,13,14,15^	2692.5 ± 202.8 *^,4,8,12,13,14,15^	271.9 ± 3.6 *^,4,8,12,13,14^

* FDR *p* ≤ 0.05 between type of Kombucha (0, 1, 7, 14 days of fermentation), *p* ≤ 0.05 between particular subgroup: ^1^—GK tea, ^2^—GK 1, ^3^—GK 7,^4^—GK 14,^5^—BK tea,^6^—BK 1, ^7^—BK 7, ^8^—BK 14, ^9^—WK tea, ^10^—WK 1, ^11^—WK 7, ^12^—WK 14, ^13^—RK tea, ^14^—RK 1, ^15^—RK 7, ^16^—RK.

**Table 2:** Statistically significant (at *p* ≤ 0.05) correlation (r) between parameters for kombucha tea * *p* value ≤ 0.05.

Correlations (r) between Analysed Parameters				
Kombucha	Green Tea	Black Tea	White Tea	Red Tea
**Time *vs***	TPC (*r* = 0.92) * FRAP (*r* = 0.73) * DPPH (*r* = −0.94) *	TPC (*r* = 0.37) * DPPH (*r* = −0.96) *	TPC (*r* = 0.89) * FRAP (*r* = 0.86) * DPPH (*r* = −0.98) *	TPC (*r* = 0.69) * FRAP (*r* = 0.62) * DPPH (*r* = −0.84) *
**Flavonoids *vs***	FRAP (*r* = −0.66) *	TPC (*r* = −0.37) * FRAP (*r* = −0.88)*	TPC (*r* = −0.43) * FRAP (*r* = −0.55) *	TPC (*r* = −0.66) * FRAP (*r* = −0.78) * DPPH (*r* = 0.56) *
**TPC *vs***	Time (*r* = 0.92) * FRAP (*r* = 0.75) * DPPH (*r* = −0.85) *	Time (*r* = 0.36) * Flavonoids (*r* = −0.36) *	Time (*r* = 0.89) * Flavonoids (*r* = −0.43) * DPPH (*r* = −0.91) *	Time (*r* = 0.69) * Flavonoids (*r* = −0.66) * FRAP (*r* = 0.87) * DPPH (*r* = −0.80) *
**FRAP *vs***	Time (*r* = 0.73) * Flavonoids (*r* = −0.65) * TPC (*r* = 0.75) * DPPH (*r* = −0.70) *	Flavonoids (*r* = −0.88) *	Time (*r* = 0.86) * Flavonoids (*r* = −0.55) * TPC (*r* = 0.93) * DPPH (*r* = −0.87) *	Time (*r* = 0.62) * Flavonoids (*r* = −0.78) * DPPH (*r* = −0.84) *
**DPPH *vs***	Time (*r* = −0.94) * TPC (*r* = −0.85) * FRAP (*r* = −0.70) *	Time (*r* = −0.96) *	Time (*r* = −0.98) * TPC (*r* = −0.91) * FRAP (*r* = −0.87) *	Time (*r* = −0.84) * Flavonoids (*r* = 0.56) * TPC (*r* = −0.80) * FRAP (*r* = −0.84) *

**Table 3:** The content of alcohol, sugar, pH and acidity in Kombucha tea. * FDR *p* ≤ 0.05 between type of Kombucha (0, 1, 7, 14 days of fermentation), *p* ≤ 0.05 between particular subgroups: ^1^—GK 0, ^2^—GK 1, ^3^—GK 7, ^4^—GK 14,^5^—BK 0, ^6^—BK 1, ^7^—BK 7, ^8^—BK 14, ^9^—WK 0, ^10^—WK 1, ^11^—WK 7, ^12^—WK 14, ^13^—RK 0, ^14^—RK 1, ^15^—RK 7, ^16^—RK 14.

Type of Beverage	Time Points (Day)	Alcohol [%]	pH	Saccharose [° Brix-g/100mL]	Acidity [mg acetic acid /L]
**Green Tea Kombucha—GK**	**0**	0.0 ± 0.00 *^,^^2,4^	5.54 ± 0.01 *^,^^2,3,4,5^	10.75 ± 0.00 *^,^^3,4^	20.12 ± 0.01 *^,^^2,3,4^
	**1**	0.2 ± 0.00 *^,^^1,3^	3.50 ± 0.04 *^,^^1,3,4^	9.75 ± 0.35 *^,^^3,4,6^	610.34 ± 0.02 *^,^^1,3,4^
	**7**	3.0 ± 0.00 *^,^^2,4^	2.61 ± 0.03 *^,^^1,2,7^	10.0 ± 0.00 *^,^^1,2,4,7,11^	7039.21 ± 0.12 *^,^^1,2,7,11,15^
	**14**	2.75 ± 0.50 *^,^^1,3^	2.49 ± 0.04 *^,^^1,2^	8.75 ± 0.00 *^,^^1,3,4^	9147.40 ± 0.31 *^,^^1,2,12,16^
**Black Tea Kombucha—BK**	**0**	0.0 ± 0.00	5.34 ± 0.03 *^,^^1,6,7,8,9,13^	11.0 ± 0.00 *^,^^6,7^	23.50 ± 0.01 *^,^^6^
	**1**	0.3 ± 0.00	3.54 ± 0.04 *^,^^5,7,8^	10.88 ± 0.18 *^,^^5,7,8,2^	501.02 ± 0.11 *^,^^5^
	**7**	3.25 ± 0.50 *^,^^6,8,3,11,15^	2.62 ± 0.03 *^,^^3,5,6,8,15^	9.5 ± 0.00 *^,^^5,6,8,3^	7039.08 ± 0.23 *^,^^6,3,11,15,^
	**14**	2.0 ± 0.00 *^,^^5,7,12,16^	2.53 ± 0.03 *^,^^5,6,7,12,16^	7.5 ± 0.00 *^,^^6,7^	9083.03 ± 0.36 *^,^^5,6,712,16^
**White Tea Kombucha—WK**	**0**	0.0 ± 0.00	6.53 ± 0.05 *^,^^5,10,11,12^	10.75 ± 0.00	21.09 ± 0.01 *^,^^10,11,12^
	**1**	0.4 ± 0.00	3.56 ± 0.06 *^,^^9,11,12^	10.13 ± 0.18	620.13 ± 0.09 *^,^^9,11,12^
	**7**	3.5 ± 0.50 *^,^^3,7^	2.53 ± 0.05 *^,^^9,10,12^	10.13 ± 0.00 *^,^^3^	7048.06 ± 0.17 *^,^^9,10,12,3,7^
	**14**	3.0 ± 0.00 *^,^^4,8^	2.37 ± 0.05 *^,^^8,9,10,11^	9.5 ± 0.00	9132.20 ± 0.43 *^,^^9,10,11,8,16^
**Red Tea Kombucha—RK**	**0**	0.0 ± 0.00	5.58 ± 0.07 *^,^^5,14,15,16^	10.75 ± 0.00 *^,^^14,15^	20.42 ± 0.03 *^,^^14,15,16^
	**1**	0.4 ± 0.50	3.62 ± 0.01 *^,^^13,15,16^	10.25 ± 0.35 *^,^^13,15,16^	600.09 ± 0.26 *^,^^13,15,16^
	**7**	3.5 ± 0.50 *^,^^3,7^	2.38 ± 0.04 *^,^^7,13,14^	10.75 ± 0.00 *^,^^13,14,16^	7059.47 ± 0.75 *^,^^7,13,14,16^
	**14**	3.0 ± 0.00 *^,^^4,8^	2.32 ± 0.02 *^,^^8,13,14^	9.5 ± 0.00 *^,^^14,15^	9071.02 ± 0.62 *^,^^4,8,13,14,15^

**Table 4:** Statistically significant (at *p* ≤ 0.05) correlation (r) between parameters for kombucha tea. * *p* value ≤ 0.05.

Correlations (r) between Analysed Parameters				
Kombucha	Green Tea	Black Tea	White Tea	Red Tea
**Time *vs***	Acidity (*r* = 0.85) * pH (*r* = 0.81) *	Acidity (*r* = 0.93) * pH (*r* = 0.96) *	Acidity (*r* = 0.99) * Alcohol (*r* = −0.88) * pH (*r* = 0.99) *	Acidity (*r* = 0.99) * Alcohol (r = −0.88) * pH (*r* = 0.86) *
**Acidity *vs***	Time (*r* = 0.85) * Alcohol (*r* = −0.61) * Saccharose (r = −0.75) * pH (*r* = 0.73) *	Time (r = 0.93) * Saccharose (*r* = −0.52) * pH (*r* = 0.88) *	Time (*r* = 0.99) * Alcohol (*r* = −0.93) * pH (*r* = 0.99) *	Time (*r* = 0.99) * Alcohol (*r* = −0.90) * pH (*r* = 0.82) *
**Alcohol *vs***	Acidity (*r* = −0.61) * Saccharose (*r* = 0.65) *	Saccharose (*r* = −0.56) *	Time (*r* = −0.88) * Acidity (r = −0.93) * Saccharose (*r* = −0.52) * pH (*r* = −0.91) *	Time (*r* = −0.88) * Acidity (*r* = −0.91) * pH (*r* = −0.65) *
**Saccharose *vs***	Acidity (*r* = −0.75) * Alcohol (*r* = −0.65) *	Acidity (*r* = −0.52) *, Alcohol (*r* = −0.56) *	Acidity (*r* = −0.52) * pH (*r* = −0.47) *	Alcohol (r = −0.72) *
**pH *vs***	Time (*r* = 0.81) * Acidity (*r* = 0.73) *	Time (*r* = 0.96) * Acidity (*r* = 0.88) *	Time (*r* = 0.99) * Acidity (*r* = 0.99) * Alcohol (*r* = −0.91) *	Time (*r* = 0.86) * Alcohol (*r* = −0.65) *

## References

[1] Blokhina O., Virolainen E., Fagerstedt K.V. (2003). Antioxidants, Oxidative Damage and Oxygen Deprivation Stress: A Review. Ann. Bot..

[2] Jakubczyk K.J.P., Piotrowska G., Janda K. (2020). Characteristics and biochemical composition of kombucha–fermented tea. Med. Ogólna Nauki Zdrowiu.

[3] Chandrakala S.K., Lobo R.O., Dias F.O., Grumezescu A.M., Holban A.M. (2019). 16—Kombucha (Bio-Tea): An Elixir for Life?. Nutrients in Beverages.

[4] Kim J., Adhikari K. (2020). Current Trends in Kombucha: Marketing Perspectives and the Need for Improved Sensory Research. Beverages.

[5] Shahidi F., Ambigaipalan P. (2015). Phenolics and polyphenolics in foods, beverages and spices: Antioxidant activity and health effects—A review. J. Funct. Foods.

[6] Jakubczyk K., Kałduńska J., Dec K., Kawczuga D., Janda K. (2020). Antioxidant properties of small-molecule non-enzymatic compounds. Pol. Merkur. Lekarski.

[7] Blanc P.J. (1996). Characterization of the tea fungus metabolites. Biotechnol. Lett..

[8] Villarreal-Soto S.A., Beaufort S., Bouajila J., Souchard J.-P., Taillandier P. (2018). Understanding Kombucha Tea Fermentation: A Review. J. Food Sci..

[9] Kapp J.M., Sumner W. (2019). Kombucha: A systematic review of the empirical evidence of human health benefit. Ann. Epidemiol..

[10] Jayabalan R., Malini K., Sathishkumar M., Swaminathan K., Yun S.-E. (2010). Biochemical characteristics of tea fungus produced during kombucha fermentation. Food Sci. Biotechnol..

[11] Ivanišová E., Meňhartová K., Terentjeva M., Harangozo Ľ., Kántor A., Kačániová M. (2020). The evaluation of chemical, antioxidant, antimicrobial and sensory properties of kombucha tea beverage. J. Food Sci. Technol..

[12] Brand-Williams W., Cuvelier M.E., Berset C. (1995). Use of a free radical method to evaluate antioxidant activity. LWT Food Sci. Technol..

[13] Pekkarinen S.S., Stöckmann H., Schwarz K., Heinonen I.M., Hopia A.I. (1999). Antioxidant activity and partitioning of phenolic acids in bulk and emulsified methyl linoleate. J. Agric. Food Chem..

[14] Benzie I.F., Strain J.J. (1996). The ferric reducing ability of plasma (FRAP) as a measure of “antioxidant power”: The FRAP assay. Anal. Biochem..

[15] Benzie I.F., Strain J.J. (1999). Ferric reducing/antioxidant power assay: Direct measure of total antioxidant activity of biological fluids and modified version for simultaneous measurement of total antioxidant power and ascorbic acid concentration. Methods Enzymol..

[16] Singleton V.L., Rossi J.A. (1965). Colorimetry of Total Phenolics with Phosphomolybdic-Phosphotungstic Acid Reagents. Am. J. Enol. Vitic..

[17] Hu S., Yuan C., Zhang C.H., Wang P., Li Q., Wan J., Chang H., Ye J., Guo X. (2013). Comparative Study of Total Flavonoid Contents from the Different Tissues and Varieties of Abelmoschus Esculentus. Int. J. Med. Sci. Biotechnol..

[18] Pękal A., Pyrzynska K. (2014). Evaluation of Aluminium Complexation Reaction for Flavonoid Content Assay. Food Anal. Methods.

[19] Gaggìa F., Baffoni L., Galiano M., Nielsen D.S., Jakobsen R.R., Castro-Mejía J.L., Bosi S., Truzzi F., Musumeci F., Dinelli G. (2018). Kombucha Beverage from Green, Black and Rooibos Teas: A Comparative Study Looking at Microbiology, Chemistry and Antioxidant Activity. Nutrients.

[20] Crafack M., Mikkelsen M.B., Saerens S., Knudsen M., Blennow A., Lowor S., Takrama J., Swiegers J.H., Petersen G.B., Heimdal H. (2013). Influencing cocoa flavour using Pichia kluyveri and Kluyveromyces marxianus in a defined mixed starter culture for cocoa fermentation. Int. J. Food Microbiol..

[21] Schlesier K., Harwat M., Böhm V., Bitsch R. (2002). Assessment of Antioxidant Activity by Using Different In Vitro Methods. Free Radic. Res..

[22] Chakravorty S., Bhattacharya S., Chatzinotas A., Chakraborty W., Bhattacharya D., Gachhui R. (2016). Kombucha tea fermentation: Microbial and biochemical dynamics. Int. J. Food Microbiol..

[23] Ettayebi K., Errachidi F., Jamai L., Tahri-Jouti M.A., Sendide K., Ettayebi M. (2003). Biodegradation of polyphenols with immobilized Candida tropicalis under metabolic induction. FEMS Microbiol. Lett..

[24] Tanaka T., Matsuo Y., Kouno I. (2009). Chemistry of Secondary Polyphenols Produced during Processing of Tea and Selected Foods. Int. J. Mol. Sci..

[25] Hur S.J., Lee S.Y., Kim Y.-C., Choi I., Kim G.-B. (2014). Effect of fermentation on the antioxidant activity in plant-based foods. Food Chem..

[26] Hosseinzadeh H., Nassiri-Asl M. (2014). Review of the protective effects of rutin on the metabolic function as an important dietary flavonoid. J. Endocrinol. Investig..

[27] Price K.R., Rhodes M.J.C., Barnes K.A. (1998). Flavonol Glycoside Content and Composition of Tea Infusions Made from Commercially Available Teas and Tea Products. J. Agric. Food Chem..

[28] ZhenJun Z., YuCheng S., HuaWei W., CaiBi Z., XianChun H., Jian Z. (2018). Flavour chemical dynamics during fermentation of kombucha tea. Emir. J. Food Agric..

[29] Shahbazi H., Hashemi Gahruie H., Golmakani M., Eskandari M.H., Movahedi M. (2018). Effect of medicinal plant type and concentration on physicochemical, antioxidant, antimicrobial, and sensorial properties of kombucha. Food Sci. Nutr..

[30] Changes in Major Components of Tea Fungus Metabolites during Prolonged Fermentation—Chen—2000—Journal of Applied Microbiology—Wiley Online Library. https://onlinelibrary.wiley.com/doi/full/10.1046/j.1365-2672.2000.01188.x.

[31] Sreeramulu G., Zhu Y., Knol W. (2000). Kombucha Fermentation and Its Antimicrobial Activity. J. Agric. Food Chem..

[32] Malbaša R.V., Lončar E.S., Vitas J.S., Čanadanović-Brunet J.M. (2011). Influence of starter cultures on the antioxidant activity of kombucha beverage. Food Chem..

[33] Chen C., Liu B.Y. (2000). Changes in major components of tea fungus metabolites during prolonged fermentation. J. Appl. Microbiol..

[34] Malbaša R.V., Lončar E.S., Djurić M. (2008). Comparison of the products of Kombucha fermentation on Sucrose and molasses. Food Chem..

[35] Greenwalt C.J., Steinkraus K.H., Ledford R.A. (2000). Kombucha, the Fermented Tea: Microbiology, Composition, and Claimed Health Effects. J. Food Prot..

[36] Jayabalan R., Malbaša R.V., Lončar E.S., Vitas J.S., Sathishkumar M. (2014). A Review on Kombucha Tea—Microbiology, Composition, Fermentation, Beneficial Effects, Toxicity, and Tea Fungus. Compr. Rev. Food Sci. Food Saf..

[37] Srinivasan R., Smolinske S., Pharm D., Greenbaum D. (1997). Probable Gastrointestinal Toxicity of Kombucha Tea. J. Gen. Intern. Med..

